# When Is a Two-Stage Surgical Procedure Indicated in the Treatment of Pseudotumors of the Hip? A Retrospective Study of 21 Cases and a Review of the Literature

**DOI:** 10.3390/jcm13030815

**Published:** 2024-01-31

**Authors:** Mariachiara Cerchiaro, Giulia Trovarelli, Andrea Angelini, Elisa Pala, Antonio Berizzi, Carlo Biz, Pietro Ruggieri

**Affiliations:** Department of Orthopedics and Orthopedic Oncology, DISCOG, University of Padova, Via Giustiniani 3, 35128 Padova, Italy; mariachiara.cerchiaro@unipd.it (M.C.); giulia.trovarelli@aopd.veneto.it (G.T.); andrea.angelini@unipd.it (A.A.); elisa.pala@aopd.veneto.it (E.P.); antonio.berizzi@unipd.it (A.B.); carlo.biz@unipd.it (C.B.)

**Keywords:** custom-made 3D-printed prostheses, hip, pseudotumors, revision surgery, total hip arthroplasty

## Abstract

(1) **Background**: A pseudotumor of the hip is a sterile, non-neoplastic soft tissue mass associated with total hip arthroplasties. Pseudotumors may mimic soft tissue tumors or infections, and thus a differential diagnosis is crucial, and biopsy is recommended. The purpose of this study was to compare the complications and functional results between one-stage and two-stage procedures. (2) **Methods**: We retrospectively analyzed 21 patients surgically treated at our institution with “pseudotumors” associated with hip prosthesis (8 male, 13 female with a mean age of 69 years). One-stage revision was performed in 10 cases and two-stage reversion in 10, with excision only in 1 case. Complications were classified as major and minor and functional results assessed using the Harris Hip Score (HHS). (3) **Results**: Five patients (24%) reported major complications. The survival rate for all complications was 75%. The overall survival rate was 95% at 5 years. The mean HHS ranged from 35 pre-op to 75 post-op, highlighting improved functional results in all cases. We recorded no differences in complications or functional outcomes between the one- and two-stage procedures. (4) **Conclusions**: In our experience, the two-stage surgical approach is preferable in cases with major bone defects and larger pseudotumor sizes. The use of custom-made 3D-printed prostheses is increasing and is a further reason to prefer two-stage revision.

## 1. Introduction

The introduction of total hip arthroplasty (THA) has become essential in treating disabilities secondary to degenerative hip joint disease to improve quality of life.

It is one of the most common orthopedic operations performed worldwide. Painful osteoarthritis of the hip is the primary indication for THA. Degenerative osteoarthritis of the hip, aside from being quite diffuse in the elder population, has a remarkable impact on quality of life. In fact, in the early stages, medical treatment is able to improve pain and function, but later on in the course of disease, impairment tends to increase, and patients who want to keep an active lifestyle have to undergo hip replacement surgery. It is also constantly growing due to the increasing age of the population. There are currently about 2.5 million people in the United States living with THA. Over 200,000 THA procedures are performed in the United States each year, and this number is expected to rise to nearly 570,000 by 2030 [1,2,3].

Over the years, several bearing surfaces have been developed: metal on polyethylene (MoP), metal on metal (MoM) ceramic on ceramic (CoC), and ceramic on polyethylene (CoP). Highly cross-linked polyethylene is now commonly used in implants and is considered the “gold standard” [4,5].

MoM THA has decreased from 20% in 2005 to <1% in 2012 [1]. The increased revision rate of MoM THA is thought to be associated with the development of unique MoM THA complications now known as adverse metal debris reactions (ARMDs), which include elevated serum metal ions, lack of ingrowth in components, aseptic loosening, metallosis, necrosis, pseudotumors, and unexplained pain [1,6].

Typically, a pseudotumor is a non-neoplastic, sterile, solid or cystic lesion of inflammatory origin, with fluid accumulation that frequently extends beyond the joint capsule [7].

The rise in THA and advancements in surgical techniques have contributed to an increase in the number of implants over the years along with associated complications such as pseudotumors. These are not uncommon, although reported incidences vary with different implants. Recent studies reported incidences ranging from 0.27% to 5% [7,8], while others reported ranges from 0.6% to 61% [9,10].

The prevalence of pseudotumors in patients with MoM hip implants varies widely in the literature. A meta-analysis reported an estimated incidence of 0.6% [11], but more recent studies have identified asymptomatic pseudotumors at rates from 31% to 60.9% [9]. Asymptomatic pseudotumors are incidental findings in 57–58% of cases [7].

Pseudotumors can be asymptomatic, but they can also cause pain, instability, and gait disturbances, leading to excision of the soft tissue mass or the need for revision arthroplasty [10].

Clinical symptoms of pseudotumors often include groin pain, skin changes or rashes, instability or spontaneous dislocation, deep vein thrombosis, hip discomfort, paresthesia, antalgic gait, and a palpable mass [7,12].

The complexity of treatment of pseudotumors lies in periprosthetic osteolysis and in extensive damage to the soft tissues, which compromise the stability of the revision implant.

The contribution of a multidisciplinary approach is evident considering the complexity of the type of intervention, especially in explant prostheses and complex prosthesis such as custom-made 3D-printed prostheses [13,14,15].

Surgery can help to restore joint stability, function, and range of motion, allowing patients to regain mobility and participate in activities they could not due to pseudotumor-related symptoms.

The two-stage approach in the treatment of pseudotumors has been scarcely analyzed in the literature.

The aim of the present study was to report the preliminary results of complex revision surgery for hip prostheses in patients suffering from “pseudotumors”, analyzing the (1) correct diagnosis, (2) appropriate treatment, with a one stage or two-stage surgical procedure, (3) incidence of complications and implant survival, and (4) functional results. We also performed a literature analysis on the diagnostic and management aspects.

## 2. Materials and Methods

### 2.1. Study Design

We conducted an observational study with a retrospective design. This single-center, comparative, clinical, and functional study included a consecutive series of Caucasian patients affected by pseudotumors of the hip and treated at our level-I healthcare trauma center (Orthopedics and Orthopedic Oncology Department, University-Hospital of Padova) from January 2016 to December 2022.

### 2.2. Patients

The following demographical and clinical data were collected for all patients included in the study, considering their sex, age at surgery, comorbidities, tribology, and time to implant revision. All patients examined came to our attention for swelling and pain in the hip and functional limitation of daily life activities.

Twenty-one patients (8 male and 13 females) with a median age of 68 years (range of 50.4–79.6 years) were included.

Fourteen patients had cardiovascular risk factors such as diabetes, hypertension, hypercholesterolemia, and obesity, while five patients had a history of tumors.

The mean time from the first implant to revision in our department was 9.86 years, with a minimum survival time for the implant of 1 year and a maximum of 20 years.

The bearing of the prosthesis was MoP in 11 cases, while 7 were MoM, 2 were CoC, and 1 was CoP.

All patients were preoperatively classified with ASA scores with a mean of 2.6 (range from 1 to 4).

They underwent plain AP-LL X-rays and second-level imaging (CT or MRI with contrast), and in all patients, we performed a biopsy to confirm the diagnosis of a pseudotumor (absence of neoplastic cells).

Two further patients out of the 21 analyzed did not undergo operations due to co-morbidities, and only an observational follow-up was maintained. These patients were excluded from the statistical analysis.

Excision was only performed in one case because this patient had recently undergone revision surgery on the prosthetic stem elsewhere, leaving the pseudotumor in place.

In 10 cases, a one-stage revision was performed, while two-stage revisions were performed in the other 10 patients. Standard prostheses were used in 6 cases, with revision prostheses in 6 cases and custom-made prostheses in 7 cases (Table 1).

### 2.3. Ethics

All subjects participating in this long-term follow-up study received a thorough explanation of the risks and benefits of inclusion and gave their written informed consent to participate in the study. This study was approved by the local ethics committee (CESC Code: 2561 P; 12 March 2012). This study was performed in accordance with the ethical standards of the 1964 Declaration of Helsinki as revised in 2013 and conducted ethically according to the most recent international standards [16].

### 2.4. Inclusion and Exclusion Criteria

Our study included adult patients, both male and female, who provided informed consent to participation in this study. The main inclusion criteria were a biopsy-confirmed diagnosis of pseudotumors obtained before surgery, a correlation with the existing prosthetic implant, and no cognitive impairment. The biopsy criteria were perivascular lymphocytic infiltrate, fibrinous exudate, macrophage accumulation, and tissue necrosis. Comorbidities that prevented surgical treatment were the exclusion criteria for this study. For this reason, 3 of the 24 patients initially considered were excluded from the study, 2 patients were advanced in age to the point of being a high operative risk, and another patient was excluded because of significant cognitive impairment.

### 2.5. Imaging

The first level of exams performed were ultrasounds with the suspicion of a mass and radiography for studying the bone. Radiographic examination is not particularly sensitive in the diagnosis of pseudotumors but can give important information regarding the quality of the bone and prosthesis. The presence of osteolysis can lead to suspicion of a pseudotumor. CT is a useful multiplanar imaging tool for evaluating hip implants. It is advantageous for assessing bone quality, heterotopic ossifications, osteolysis, and metallosis. CT allows imaging of radiographically occult cystic and solid pseudotumors, although it is less sensitive than MRI with contrast for evaluating adverse local soft tissue reactions. The administration of iodinated contrast medium is useful for vascular studies and localization and characterization of periarticular cystic pseudotumors [7]. Magnetic resonance imaging (MRI) is an excellent modality for evaluating periarticular soft tissue complications after hip arthroplasty. Pseudotumors have a variable appearance on MRI, as alterations shown on the T1-T1 signal may mimic soft tissue tumors with no clear margins. Pseudotumors range from discrete thin-walled cystic lesions to ill-defined solid masses, often associated with synovial thickening, surrounding fluid, or scattered debris [7,17]. Clinical presentation may be similar to an infection, and thus a differential diagnosis is crucial as it may be symptomatic (more or less) or asymptomatic.

### 2.6. Biopsy

Histologic evaluation with biopsy is recommended and, in our experience, mandatory for excluding diagnoses of tumors or infection.

When a pseudotumor is suspected, a biopsy is required for differential diagnosis with malignant tumors in bone and soft tissue such as liposarcoma, synovial cell sarcoma, malignant peripheral nerve sheath tumor, non-Hodgkin’s lymphoma, osteosarcoma, and chondrosarcoma.

Among the benign masses, we find seromas and hematomas, which are frequent complications of hip arthroplasty. Seroma and hematoma are differentiated from a pseudotumor by their development in the immediate postoperative period and subsequent resolution over time.

An infection is also differentially diagnosed with pseudotumors, but an infection often includes local or systemic symptoms and signs such as fever, palpable thermotact-positive mass, and flushing. Peripheral enhancement of the cystic fluid collection is the typical finding in post-contrast imaging.

Close communication between physicians, radiologists, and pathologists is required to ensure accurate interpretation of biopsy results [7,18].

### 2.7. Histology

Pseudotumors are histologically described as aseptic lymphocytic vasculitis-associated lesions [19]. These tissue reactions can manifest as an effusion, local tissue necrosis, or periprosthetic osteolysis which may be solid or cystic [20]. Histopathologically, pseudotumors are described as cell-mediated (type IV) hypersensitivity reactions characterized by perivascular lymphocytic infiltrate, fibrinous exudate, macrophage accumulation, and tissue necrosis [12,21].

### 2.8. Surgical Procedure

The surgical procedures performed were excision of the tumor or excision and revision of the implant, and these could be carried out in different ways: with a standard prosthesis, a revision prosthesis, or a custom-made prosthesis, which was used when the size of the bone loss precluded the use of a standard or revision prosthesis.

Excision and revision could also be performed in two different ways: one-stage or two-stage surgery. The choice of which strategy to use was based on (1) the general condition of the patient; (One-stage surgery requires hemodynamic stability of the patient.) (2) the size of the pseudotumor, (If the tumor is large with bone erosion, then the two-stage surgery is variable.) and (3) the type of implant, because with custom-made 3D prostheses, which require a high-resolution CT, the imaging study is better when performed after removal of the implant to allow assessment of the bone loss and planning of the implant.

**Figure 1:**
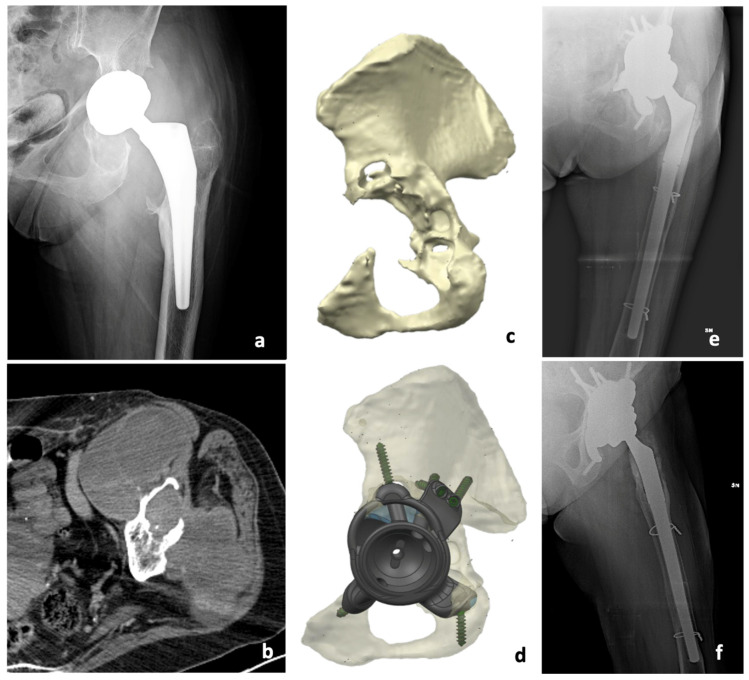
Imaging studies and surgical steps from a patient with pseudotumor of the hip treated with a two-stage procedure using an acetabulum custom made 3D printed. Preoperative X-ray (**a**) and CT scan (**b**) showing pseudotumor, as well as 3D CT scan performed after the first surgery (**c**) and 3D-printed model of the acetabular (**d**) with postoperative X-rays (**e**,**f**).

In our study, in 6 cases, we used a standard revision prosthesis, with total hip prostheses in 4 cases and only the acetabular component in 2 cases. In 7 cases, we used a custom-made prosthesis because of bone loss (Figure 1 and Figure S1). We aimed to investigate if there were differences in terms of complications and functional results between the types of surgery.

When feasible, we tried to use the preexisting surgical approach, enlarging it if necessary. Therefore, in most of these revision cases, a later or posterolateral approach was used. In those more difficult cases requiring approaching the inner pelvis in order to access the major vascular axis to control bleeding or remove large pseudotumors abutting or frankly extending medially to the iliac bone, an Enneking approach was combined with the lateral approach to the hip.

### 2.9. Postoperative Protocols of Both Procedures

The time of admission for surgery varied from 9 to 40 days, depending on the type of surgical treatment. All patients followed the same postoperative protocol in the same standardized manner. Patients were administered preoperative intravenous antibiotics and continued to receive antibiotics for 7 days. Postoperative management included bed rest, analgesia, and mobilization with a walker or crutches after the second postoperative day when possible. Chemical anti-thrombosis prophylaxis was given until complete weight-bearing with low molecular weight heparin. All patients received physical therapy for a minimum of 6 weeks after being discharged from the hospital. Immobilization was obtained with a pelvic thigh brace in all cases of pelvic reconstruction with custom-made prostheses and in all cases of complex revisions. The brace was positioned at 10° of abduction, fixed in extension for 1 month, and then unlocked, with flexion allowed up to 90° for another month and walking with two crutches, with a progressive load in the second month.

### 2.10. Clinical and Radiographic Evaluation

Patients had plain X-rays (AP-LL) and second-level imaging (CT or MRI with contrast imaging), and in all patients, we performed a biopsy to confirm the absence of neoplastic cells and support the diagnosis of a pseudotumor. Preoperative radiographic data were obtained from the anteroposterior and lateral views of the hip and femur. The bone–implant interface and migration of the acetabular component in the horizontal and vertical directions were examined in the immediate postoperative and final follow-up radiographs.

We attempted to stratify our cases according to the severity of the bone defects evaluated with the Paprosky classification as well as the size of the pseudotumor tissue using an ideal axial diameter [22].

The modified Harris Hip Score (HHS) was used for the clinical evaluations of all patients at each check (1969, Harris WH). Every patient was evaluated clinically with a questionnaire and with the Harris Hip Score (HHS), with a maximum score of 100. A result over 70 points indicates a good state for the hip articulation [23]

### 2.11. Complications

Complications were analyzed according to the classification by Henderson et al. (2011–2013). Endoprosthesis failures were classified as soft tissue failures (type I), aseptic loosening (type II), structural fractures (type III), or infections (type IV). Tumor recurrence (type V) was not considered because this was an exclusion criterion for our study.

The complications were then divided into major and minor, where among the major ones were deep periprosthetic infections requiring revision and death [24].

### 2.12. Statistical Analysis

Before handling, the data were preprocessed and visually inspected for quality control, missing data, and potential outliers. The normality of the data distribution was verified by conducting a Shapiro–Wilk test, which was preferred over other tests (including the omnibus test of Kolmogorov–Smirnov or D’Agostino–Pearson) due to the small sample size employed. Continuous variables were expressed as a mean and range (minimum and maximum), while categorical parameters were calculated as percentages where appropriate. The Student’s *t*-test for paired samples was used to compare the pre- and postoperative HHS values, and the Student’s *t*-test for independent samples was used to compare the postoperative HHS values between the one-stage and two-stage procedures and HHS improvement between the one-stage and two-stage procedures. A scatterplot was used to see the correlation between the age of pseudotumor onset and age and to assess the pre- and postoperative HHS values in relation to age and sex.

The survival of the prosthesis through complications was evaluated with Kaplan–Meier curves (1958, Kaplan EL). Finally, Cox proportional hazard regression and the Longrank test were used to compare the complications and implant survival between the one-stage and two-stage surgical procedures. Statistical significance was defined as *p* ≤ 0.05. Statistical analysis was performed using MedCalc software Version 22.018. (MedCalc Software, Broekstraat 52, Mariakerke, Belgium) [25].

## 3. Results

### 3.1. Patients’ Data

**Figure 2:**
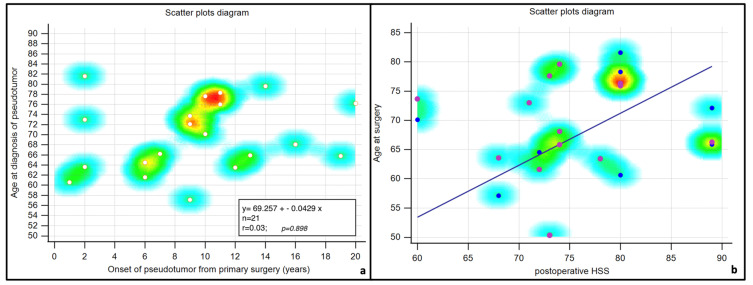
Scatter plots. Correlation between age at diagnosis of the pseudotumor and years from primary surgery (**a**) and between age at surgery and postoperative Harry Hip Score (**b**). In both cases, there were no statistically significant differences. Colors indicate scatter plot data density decreasing from red to light blue.

From the distribution of data, the pseudotumors in our population arose between 8 and 12 years after implantation of the prosthesis and, for the most part, in subjects above 70 years of age. The scatter plot used to see the correlation between age and pseudotumor onset in relation to age shows that there were no statistically significant differences in age (Figure 2).

There were no significant differences between sex and age in the postoperative functional outcomes. The biopsies showed chronic inflammation, giant cells, lymphocytic infiltration, metallosis, necrosis, and fibrous tissue.

Our patients were stratified according to the Paprosky classification of bony defects. Pseudotumors do not fit perfectly into this classification of bony defects since we also must consider the extension and location of the pseudotumor itself, which have a remarkable role in the choice of and indication to surgery. As far as acetabular defects are concerned, we found that 5 cases were Paprosky type I, 10 cases were Paprosky type II, and 6 were Paprosky type III.

As far as femoral defects were concerned, our series included 10 patients for type I, 4 patients for type II, 5 patients for type III, and 2 patients for type IV. The acetabular and femoral defects according to Paprosky classification are summarized in Table 2 showing that acetabular and femoral defects were usually combined and that cases with a “major” combined Paprosky classification (acetabular type III and femoral type III or IV) were mostly treated with a two-stage procedure (Table 2).

Moreover, the entity of the bony defect was not the sole factor for deciding between one-stage and two-stage procedures, since the size, extension, and location of the pseudotumor itself also contribute to this choice. In fact, we stratified our cases into two groups according to size of the pseudotumors below and above an average size (ideal axial diameter) of 7 cm. Here, 10 cases out of 14 had an axial size of 7 cm, and more had two-stage surgical procedures, while all 6 cases with a pseudotumor axial size below 7 cm had one-stage procedures. One remaining case only had the pseudotumor excised in our hospital, since the revision of prosthesis had already been performed elsewhere previously (Table 2).

Clinical evaluation of our patients showed that the mean preoperative HHS was 35 (range: 18–45), indicating a relevant compromission of the joint in all subjects. The mean postoperative HHS was 75 (range: 60–89). In our study population, there was a statistically significant difference between the postoperative and preoperative HHS values, as confirmed by a paired sample t-test (*p* < 0.0001). When comparing the one- and two=stage procedures, there were no statistically significant differences in the postoperative HHS (*p* = n.s.) and HHS improvement (*p* = n.s.) (Table 3).

### 3.2. Complications

**Figure 3:**
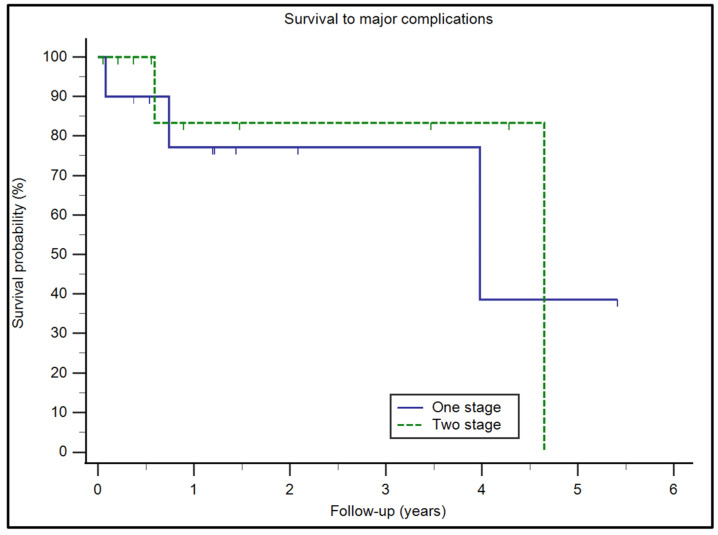
Kaplan–Maier survival curve. Survival through major complications is compared between one- and two-stage procedures.

The complications in our study at a mean follow-up of 20 months (range: 4–65 months) were reported in 8 patients (8/21 patients, 38.1%): 2 were wound dehiscence (type I) within the first 30 days after surgery, which was treated with wound revision and antibiotics. One case of a thigh sensory deficit was found, improving 6 months after surgery. Five major complications were found: two deaths at 3 months and 1 year after surgery, and three patients reported periprosthetic infection (type IV) treated with surgical debridement and antibiotic therapy. In one patient, for the isolation of *Proteus mirabilis* and *E. coli*, surgical cleaning was performed, covering the prosthesis with a rectus abdominis flap. None of our patients had aseptic loosening or implant breakage (type II and III, respectively). The survival rate of major complications at an average follow-up of 20 months (range: 4–40) was 75%, as represented by the Kaplan–Maier curves (Figure 3). The overall survival rate was 95% at 5 years. No statistically significant differences in the complication rates (*p* = 0.7521) were found between the one-stage and two-stage procedures, as demonstrated by a comparison of the survival curves (logrank test). We registered urinary tract infections in three patients. However, considering that they were managed with standard antibiotic therapy without any further complications related to the prosthesis, they were not included in the complications. Cognitive impairment was an exclusion criterion from our study, since a patient with cognitive impairment is uncooperative and cannot benefit from such a demanding surgery. We did not observe delirium in our patients. On average, the patients received two units of concentrated red blood cells during hospitalization. For those undergoing major revision surgeries, additional perioperative transfusions (up to six) were required. The results are summarized in Table 4.

## 4. Discussion

### 4.1. Diagnosis

A pseudotumor of the hip refers to a non-neoplastic soft tissue mass that can develop in the region around the hip joint. The term “pseudotumor” is used because the mass can mimic a tumor in terms of its appearance and growth pattern, but it is not a true tumor. For this reason, a biopsy is mandatory. In our study, all patients underwent biopsies.

Pseudotumors around the hip joint are often caused by an inflammatory reaction to wear debris from the components of a hip implant. This condition is most commonly associated with MoM hip joint replacements. In our study, 13 (61.9%) women were affected by pseudotumors, as well as 8 males (38.1%). Grote et al., in their literature review of pseudotumors arising in MoM implants, pointed out that women are at increased risk of complications from THA, possibly related to metal hypersensitivity [1].

Hasegawa et al. in their study described more pseudotumors being present in women after MoM THA, but several case reports on pseudotumors in males or females from MoM implants are described in the literature. Moreover, the implant type and implant malposition are most often involved in pseudotumor formation [26]. As reported in the literature by various studies, pseudotumors are recorded in MoM implants and less so in CoC implants [8,27,28].

In our study of 21 patients with pseudotumors, there were 11 (52.3%) MoP implants followed by 7 (33.3%) MoM implants, 2 (9.5%) CoC implants, and 1 (4.7%) CoP implant. Only two cases of pseudotumors were recorded for CoC implants, in line with the literature [8,27,28].

In the past, pseudotumors were mainly related to MoM hip articulations, but considering that MoP implants are the most used type nowadays, a high frequency of pseudotumors was observed in MoP implants in our study as well as in previous studies [29]. A difference between the pseudotumors associated with MoM, which are usually inflammatory, and those associated with MoP implants, which are usually fibro-necrotic, was highlighted in studies by Felipe et al. and Hjorth et al. [29,30].

The data collected from the various studies are summarized in Supplementary Table S1 [31,32,33,34,35,36,37].

This study, in accordance with the current literature, was carried out with regard to sex, age of onset, and time of symptom onset. Symptoms may present with the appearance of soft tissue masses from 3 to 26 years after implantation. In our study, the mean onset of symptoms was 9.8 years.

In a 2016 study, Hasegawa et al. described several types of mixed and solid cystic-type pseudotumors [26].

In the literature, risk factors for the formation of a pseudotumor are high serum cobalt (Co >5 μg/L) and chromium (Cr) levels (known to cause osteolysis), being female [7], a high cup inclination angle > 55°, and a large diameter head in THA > 36 mm [1,38,39,40,41]. Despite the observed associations and risk factors, the exact mechanism of THA-induced pseudotumors is still unclear [42]. It is well established that various surfacing couplings significantly contribute to wear, generating particles that serve as the foundation for a reactive and granulomatous process and histologically forming a pseudotumor. As reported above, different prosthetic surfaces may have different impacts on pseudotumor pathogenesis, but none of these surfaces are safe from pseudotumor formation. For the other mentioned risk factors, such as sex, inclination angle, and head diameters, their roles are less clear, and they have been gleaned from the existing literature [42].

### 4.2. Treatement

These data were not taken into consideration by our study to focus instead on surgical indications.

Our patients were all symptomatic, and on the contrary, asymptomatic patients with pseudotumors are described in the literature. Hasegawa et al. in their study described how pseudotumors of mixed compositions are associated with pain, whereas cystic pseudotumors are mostly asymptomatic [26]. In contrast, a study by Hart et al. found no correlation between the type of pseudotumor and the presence of symptoms [42].

In a study by Lindsay et al., the authors showed that there is no correlation between pseudotumor size and a patient’s symptoms [9]. In our study, the patients had hip pain and swelling and not nerve or vascular deficits. Sagoo et al. described a symptomatic MoM pseudotumor with extension from the hip joint to the iliopsoas mass in the pelvis, with acute lower abdominal pain and motor deficits [43].

In our study, we matched two cases of a pseudotumor in a CoC prosthesis. Few cases are described in the literature. Rodriguez described a pseudotumor in a CoC prosthesis in which ceramic wear debris could lead to an ALTR and nerve complications [44].

In the literature, the controversy about whether to pursue surgery for pseudotumor treatment revolves around various factors, including the patient’s symptoms, the severity of the pseudotumor, the potential risks and benefits of surgery, and the overall health of the patient. There is not a one-size-fits-all solution, as treatment decisions should be tailored to each individual’s unique situation. Surgery can provide effective relief from pain, discomfort, and other symptoms associated with pseudotumors, especially if the condition is causing significant impairment to daily activities and quality of life.

In light of what was reported above, we feel that surgery of pseudotumors is recommended in all cases where the pseudotumor and related changes in implant stability are symptomatic and progressively increasing in an otherwise active patient. Surgery is certainly recommended for larger pseudotumors and symptomatic pseudotumors, and it also has to be considered when there are elevated serum cobalt and chromium levels; otherwise, a progressive increase in these levels may cause further morbidities. Surgical recommendations include that an adequate prosthetic implant revision needs to be associated with the removal of the pseudotumor. This can be obtained in a single- or two-stage procedure. The choice between a one- or two-stage procedure is further discussed in the following paragraph, as it is related to complications.

### 4.3. Complications

Pseudotumors can lead to complications like implant loosening, tissue damage, and joint instability.

The correlation between pseudotumors and aseptic loosening needs to be further investigated since aseptic loosening may cause debris formation, favoring pseudotumor formation. Conversely, it is also true that the inflammatory and granulomatous tissue that constitute a pseudotumor is able to favor or implement aseptic loosening. Therefore, this is a quite reciprocal interaction that characterizes the pathogenesis of pseudotumors and certainly needs to be studied further. This interaction is relevant to the indications for surgery. To radically solve this problem, it is necessary to both remove the pseudotumoral tissue and revise the loosened implant.

Surgery can address these issues and prevent further damage, potentially leading to better long-term outcomes. Surgery may help restore joint stability, function, and range of motion, allowing patients to regain mobility and participate in activities they could not previously due to pseudotumor-related symptoms. Surgery, like any medical procedure, carries risks such as infection, bleeding, and anesthesia complications. Surgery often requires a period of recovery and rehabilitation, which can be demanding for patients. Not all pseudotumors cause significant symptoms or complications. In cases where the pseudotumor is asymptomatic or causing only minor discomfort, a “watchful waiting” approach might be considered to avoid unnecessary surgery. Some patients may have medical conditions that give surgery a higher risk. In such cases, the potential benefits of surgery need to be carefully weighed against the patient’s overall health [8,10].

Davis et al. in their review article stated that pseudotumors without associated pain, disfunction, or elevated metal ion levels are more likely to receive continuous surveillance without surgery [7].

Filer et al. reported the case of a hemorrhagic pseudotumor that can be successfully managed conservatively. This patient reported no trauma, was not taking anticoagulants, and had no bleeding disorders. The rapid progression in size of the pseudotumor caused significant symptoms and functional impairment. This case presented a significant clinical challenge in decision making regarding appropriate management [10].

In our study, we only analyzed operated patients.

The decision to pursue surgery for pseudotumor treatment should be made in close consultation and with a multidisciplinary approach. There are several reasons supporting a multidisciplinary approach in the management of many orthopedic diseases. First of all, a close collaboration in the diagnosis between a surgeon, radiologist, and anesthesiologist may provide a better evaluation of risks and a safer preparation of patients for surgery. Second, at the time of surgery, a close collaboration and involvement of vascular surgeons (when needed) and plastic surgeons (most of the time) with the orthopedic surgeon is useful to reduce the risk of complications and morbidity from the surgery itself. In the postoperative setting, the orthopedic surgeon collaborates with the rehabilitation doctor and professionals to give the best possible advantage to the patient in his or her recovery [45,46].

A multidisciplinary approach can analyze a situation from different points of view and has numerous advantages which can be summarized as follows: (1) providing different perspectives for the same clinical problem, (2) a combined clinical approach, (3) creating comprehensive research questions, and (4) developing definitions and guidelines. Effective collaboration between healthcare professionals significantly improves mutual knowledge and trust with improved results for both patients and providers. In recent decades, the collaboration has become wider, especially in surgery, adding plastic and vascular surgeons, general surgeons, and microsurgeons [45].

One-stage or two-stage surgery is controversial in the treatment of pseudotumors.

In the one-stage approach, the removal of problematic hip implants, pseudotumors, and revision implants is performed in a single surgical procedure. This approach is chosen when the pseudotumor is causing significant symptoms or complications and is generally preferred when the extent of tissue damage is limited and the patient’s overall health permits more extensive surgery. The procedure involves removing the problematic implant, cleaning out any inflammatory tissue, and potentially replacing the implant with a different type of implant that is less likely to cause an inflammatory response. The two-stage approach involves two separate surgical procedures. In the first stage, the failed hip implant is removed, and the pseudotumor and inflammatory tissues are cleared. The joint can be left without an implant temporarily to allow for reduction of the inflammatory response, tissue healing, and planning a revision prosthesis. After a period of several months, a second surgery is performed to place a new, more appropriate hip implant. In recent years, 3D printing applications for titanium have become available. This topic is closely related to computer-assisted surgery (CAS) and the optimization of data derived from preoperative imaging studies for the improvement of clinical and surgical outcomes, such as the accuracy of bone cuts. The use of 3D-printed prostheses is increasing, especially in musculoskeletal oncology as well as in cases of complex revisions with extensive bone loss. Improving 3D printing technology allows for the creation of custom implants to face complex reconstructions [14,15].

In our study, a one-stage surgical procedure was performed for 10 patients: 6 patients with standard prostheses, 3 with revision prostheses, and 1 with a custom-made 3D-printed prosthesis. The two-stage surgical procedure was instead performed in six patients with revision custom 3D-printed prostheses and four with revision prostheses. We recorded no differences in terms of complications or functional outcomes between the two interventional procedures. Only in one case did we perform excision of the pseudotumor without revision of the implant performed due to a recent revision surgery performed elsewhere.

There are few studies in the literature reporting on two-stage procedures.

Cottino et al. reported the case of a 72 year-old woman with a cystic pseudotumor on MoM THA. This case was treated in a two-step procedure, emphasizing the importance of careful planning for the type of surgery to be performed [47]. Moreover, the authors underlined how a two-stage revision surgery should be considered in cases of huge cystic pseudotumors to avoid rupture of the cyst and facilitate the revision procedure, reducing surgical times and thus decreasing the risk of infections and bleeding [47].

However, in a study by Sagoo et al., the authors described the excision of a pseudotumor in one stage, which led to a marked improvement in symptoms [43].

In our study, in cases with large bone loss of substance, we preferred a two-stage procedure, reducing the surgical time for each surgery and being able to better plan a revision implant.

In our study, we had no prosthetic dislocations. In the literature, Huang et al. in their case report described a case of dislocation in a 73-year-old woman after removal of a pseudotumor for a CoC prosthesis [37,48].

The 21 patients analyzed found benefits from the surgery, with functional improvement measured as a statistically significant difference between the pre- and postoperative HHS values (*p* < 0.0001). Additionally, we observed no difference in improvement and postoperative HHS between the one-stage and two-stage procedures. All enrolled patients were operated on in the same center and followed according to a standardized institutional postoperative protocol, reducing the confounding bias. Due to the rarity of the lesion and the small number of patients in related studies, quantitative functional measures were rarely used. As explained below, a possible bias may exist due to the fact that the two-stage procedure has been preferably performed for larger pseudotumors.

In our study, major complications involved eight patients: two of them died 3 months and 1 year after surgery, and five had deep infections There were no statistically significant differences in the complications between the one-stage and two-stage procedures (*p* = 0.7521). There are few data in the literature regarding complications from pseudotumor surgery, and everyone agrees on a quite high complication rate [47].

We preferred a two-stage surgical approach in cases with the association of “major” combined Paprosky classifications (acetabular type III and femoral type III or IV) and pseudotumor sizes of 7 cm or above. The Paprosky classification itself is not sufficient for the indication of treatment since the size and location of a pseudotumor must also be considered. A comprehensive classification that can take into account both the entirety of the bony defects and the extension and location of the pseudotumor is still lacking, and future studies on broader patient populations will be required to propose such a classification and define robust treatment recommendations.

The main limitation of our study was the small sample size, which was mainly due to the rarity of the investigated condition.

## 5. Conclusions

In suspected pseudotumors, a biopsy is always recommended. Given the rarity of the disease, the choice of surgical treatment remains controversial. Surgery is recommended for symptomatic pseudotumors, large pseudotumors, or the presence of elevated serum cobalt (Co > 5 μg/L) and chromium (Cr) levels. The first two clinical criteria mentioned for surgical indications far outweigh the importance of ion concentration levels, since these are often variable in symptomatic pseudotumors. Prosthetic revision associated with the removal of pseudotumors can be performed in a single or two-stage procedure.

We recorded no differences in complications or functional outcomes between the one- and two-stage procedures. In our experience, a two-stage surgical approach is preferable in cases with the association of “major” combined Paprosky classification defects and pseudotumor sizes of 7 cm or above.

Finally, the use of custom 3D-printed implants is increasing in these cases, and this is a further reason to prefer two-stage procedures.

## Figures and Tables

**Table 1:** Demographic and clinical characteristics in the cohort of patients.

Variable	Patients (n = 21)
Age (at surgery)	69 range (50–82)
Sex - Male - Female	8 13
Tribology - MoM - MoP - CoC - CoP	7 11 2 1
Time to revision	10 (yrs) range (1–20)
Prostheses - Standard - Revision - Custom-made 3D-printed	6 7 7
Reconstruction - One stage - Two stage - Excision only	10 10 1
Comorbidities - Diabetes mellitus - Hypertension - Atrial fibrillation - Hypercholesterolemia - Dyplasia - Others (stoke, arthritis, obesity, dysplasia, etc.)	4 12 2 3 2 9

Abbreviations: MoM = metal on meal; MoP = metal on polyethylene; CoC = ceramic on ceramic; CoP = ceramic on polyethylene; yrs = years.

**Table 2:** Paprosky classification of bony defects and pseudotumor size.

Patient	Previous Revision Surgery	Paprosky Classification, Acetabular	Paprosky Classification, Femur	Pseudotumor Size (Axial × Lateral) (cm)	Surgery
1 (V.G.)	Yes	I	IV	8 × 10	One-stage
2 (A.B.)	no	I	II	2 × 2	One-stage
3 (G.I.)	no	II	I	4 × 6	One-stage
4 (L.C.)	no	II	I	5 × 6	One-stage
5 (M.F.)	yes	I	I	5 × 5	One-stage
6 (L.P.)	no	III	III	10 × 9	Two-stage
7 (L.V.)	yes	III	I	10 × 7	Two-stage
8 (E.S.)	no	III	III	9 × 10	Two-stage
9 (G.B.M.)	yes	I	I	6 × 7	Excision only
10 (A.I.)	yes	II	III	12 × 15	Two-stage
11 (K.M.V.)	yes	III	IV	11 × 20	One-stage
12 (M.A.M.)	no	II	II	5 × 5	One-stage
13 (M.P.)	no	II	II	10 × 17	Two-stage
14 (M.G.G.)	yes	III	I	10 × 9	Two-stage
15 (Z.L.)	no	I	III	7 × 10	Two-stage
16 (D.G.)	no	II	III	7 × 10	Two-stage
17 (F.S.)	no	II	I	9 × 6	One-stage
18 (A.F.)	no	II	II	7 × 7	Two-stage
19 (M.D.)	yes	III	I	9 × 9	Two-stage
20 (G.C.)	no	II	I	5 × 8	One-stage
21 (G.L.)	no	II	I	9 × 10	One-stage

**Table 3:** Pre- and postoperative HHS and improvement.

Patient	HHS Preoperative	HHS Postoperative	Improvement HHS	*p* Value
1 (V.G.)	41	89	48	
2 (A.B.)	45	60	15	
3 (G.I.)	33	74	41	
4 (L.C.)	25	71	46	
5 (M.F.)	32	72	40	
6 (L.P.)	42	80	38	
7 (L.V.)	39	72	33	
8 (E.S.)	42	74	32	
9 (G.B.M.)	43	80	37	
10 (A.I.)	18	68	50	
11 (K.M.V.)	36	73	37	
12 (M.A.M.)	29	78	49	
13 (M.P.)	27	73	46	
14 (M.G.G.)	36	80	44	
15 (Z.L.)	25	60	35	
16 (D.G.)	25	68	43	
17 (F.S.)	33	74	41	
18 (A.F.)	36	80	44	
19 (M.D.)	41	89	48	
20 (G.C.)	41	89	48	
21 (G.L.)	39	80	41	
Mean	35	75	41	*p* < 0.0001

HHS = Harris Hip Score. Comparison between preoperative and postoperative HHS performed using Student’s *t*-test for paired samples.

**Table 4:** Surgical procedures and complications.

Patient	Surgery	Tribology	Complications (According to Henderson)	Minor	Major
1 (V.G.)	One-stage	MoP	IV		Deep infection
2 (A.B.)	One-stage	MoM			
3 (G.I.)	One-stage	MoP			
4 (L.C.)	One-stage	MoP			
5 (M.F.)	One-stage	MoP			
6 (L.P.)	Two-stage	MoP			
7 (L.V.)	Two-stage	MoP	IV		Deep infection
8 (E.S.)	Two-stage	MoM			
9 (G.B.M.)	Excision only	MoP			
10 (A.I.)	Two-stage	MoP	I	Superficial infection	Death at 3 months
11 (K.M.V.)	One-stage	MoM	IV		Deep infection
12 (M.A.M.)	One-stage	MoM			Death at 1 year
13 (M.P.)	Two-stage	MoM			
14 (M.G.G.)	Two-stage	CoP			
15 (Z.L.)	Two-stage	MoM			
16 (D.G.)	Two-stage	MoM	I	Superficial infection	
17 (F.S.)	One-stage	MoP			
18 (A.F.)	Two-stage	MoP			
19 (M.D.)	Two-stage	MoP	I	Femoral nerve injury, sensosy deficit	
20 (G.C.)	One-stage	CoC			
21 (G.L.)	One-stage	CoC			

Abbreviations: MoM = metal on meal; MoP = metal on polyethylene; CoC = ceramic on ceramic; CoP = ceramic on polyethylene; yrs = years.

## Data Availability

The dataset supporting the conclusions of this review is available upon request from the corresponding author.

## Supplementary Materials

The following supporting information can be downloaded at https://www.mdpi.com/article/10.3390/jcm13030815/s1. Figure S1: Case of two-stage procedure. Table S1: The published literature on management of pseudotumors of the hip [1,8,10,12,26,27,31,32,33,34,35,36,39,42,43,44,47,48,49,50,51,52,53,54,55,56,57,58].
