# Supplementary material for: When Is a Two-Stage Surgical Procedure Indicated in the Treatment of Pseudotumors of the Hip? A Retrospective Study of 21 Cases and a Review of the Literature
Source: J Clin Med. 2024 Jan 31;13(3):815. doi: 10.3390/jcm13030815 (PMC10856725; doi:10.3390/jcm13030815)
Supplement: Supplementary file 1 [file jcm-13-00815-s001.zip › Supplementary_material/Figure_S1_Legend.pdf]

Figure S1. Example of two- stage surgical procedure. Preoperative X-ray (a) and CT scan (b) showing pseudotumor. First stage of the procedure (c). 3D CT scan performed after the first surgery and 3D printed model of the acetabular (d). Custom-made acetabular (e). Second stage of the procedure (f,g). Post operative X-rays (h,i).
