# Supplementary material for: When Is a Two-Stage Surgical Procedure Indicated in the Treatment of Pseudotumors of the Hip? A Retrospective Study of 21 Cases and a Review of the Literature
Source: J Clin Med. 2024 Jan 31;13(3):815. doi: 10.3390/jcm13030815 (PMC10856725; doi:10.3390/jcm13030815)
Supplement: Supplementary file 1 [file jcm-13-00815-s001.zip › Supplementary_material/Table_S1 DEF.pdf]

Table S1. Published literature on management of pseudotumors of the hip.

Authors	Study	N° patients	Age(mean)	Time to revision	Comorbidity	Gender	Byopsy pre	Tribology	Symphthoms	Surgery	Complications	Outcomes
Pandit et al. (2008) [49]	Retrospective study	17	53 (35 to 73)	12	N.R	F		MoM	Pain dislocation nerve palsy	One stage	no	Synmptoms improved
Maurer et al. (2011) [33]	Case report	1	38	1	N.R	F		MoM	N.R	One stage (CoC	N.R	Complete Resolution symptomsafter 20 months
Parfitt et al.(2012) [34]	Case report	1	64	1.4	N.R	M		MoM	N.R	One stage (MoP	N.R	Good functional
Algarni et al.(2012) [35]	Case report	1	54	5	N.R	F		MoM	N.R	One stage (CoC	N.R	Complete Resolution symptomsafter 6 months, no recurrance
Memon et al.(2013) [36]	Case report	1	54	5	N.R	F		MoM	N.R	One stage (CoC	N.R	Good function
Kawakita et al.(2013) [37]	Case report	1	69	1.2	N.R	F		MoM	N.R	One stage (CoC	N.R	Good function after 3 months
Almousa et al. (2013) [50]	Prospective study	20	61	N.R	N.R	12F 8 M		13 MoM 3 MoP	N.R	5 patients revision	no	instability Femoral nerve palsy slowly resolves
Bisschop et al. (2013) [51]	N.R	40	N.R	N.R	N.R	N.R		N.R	11 Symptomatic 29 asymptomatic	N.R	N.R	N.R
Nawabi et al. (2013) [52]	N.R	55	N.R	N.R	N.R	N.R		N.R	40 Symptomatic 15 asymptomatic	N.R	no	N.R
Fu et al (2015) [53]	Case report and review	2	60 (1) 47 (2)	20 (1) 28(2)	Ankylosing spondylitis Ankylosing spondylitis	M M		MoP MoP	yes	Swelling pain Swelling pain	Emipelvic amputation One stage (THA revision)	no
Zhai et al. (2015) [54]	Retrospective study	8	N.R	31.9(range 18-45)	Hemophilia A(7) B(1)	M		N.R	N.R	N.R	Pseutotumor excision and fixation	3 infection
Abdel-Hamid et al.(2015) [38]	Case report	1	75	6	N.R	F		MoM	N.R	One stage (CoP	N.R -	Significant improvement of swelling after 9 months
Hasegwa et al (2016) [28]	Retrospective study	62	64	45.7 months symptomatic 49.7 months asymptomstic	N.R	49 F 13 M		MoM	16 Symptomatic (larger pseudotumor) 46 asymptomatic	N.R -	N.R	N.R
Campbell et al. (2016) [8]	Case report	1	57	9	N.R	F		CoC	pain	One stage	no	asyntomatic
Cottino et al. (2017) [17]	Case report	1	72	15	N.R N.R	F		MoM	Abdominal mass, pain	Two stage	no	Pain free
Blau et al. (2017) [12]	Case report	1	69	7	N.R.	F		CoM	Hip pain 8 elevated serum cobalt	N.R	no	No recurrence decresed level cobalt chromium

N.R.: not reported, M:male; F:female; MoM: Metal on metal; MoP:Metal on Polyetilene; CoC: Ceramic on Ceramic; CoM: Ceramic on Metal; THA: total Hip Artroplasty

Authors	Study	N° patients	Age(mean)	Time to revision	Comorbidity	Gender	Tribology	Biopsy pre	Symphthoms	Surgery	Complications	outcomes
Konan et al. (2017) [55]	Compparative study, ultrasound in pseudotumor	71	56(34-38)	N.R	N.R	24 F 47 M	MoM		15 asyntomatics 8 syntomatics	8 patients revision THA syntomatic pseudotumor	N.R	N.R
Sutphen et al. (2018) [56]	Retrospective study	70	N.R	N.R	N.R	N.R		N.R	31 Symptomatic 39 asymptomatic	N.R	N.R	N.R
Smeekes et al. (2018) [41]	Comparison study of three grading system with MRI		N.R	N.R	N.R		MoM		54 symptomatic 52 asymptomatic	N.R	N.R	N.R
Gudda et al. (2018) [29]	Case report	1	50	8	N.R	F	CoM		pain	One stage	no	Good result HHS pre op 58 post op 87
Persson et al. (2018) [57]	Prospective study	13	66	6,7 (2-16)	osteoarthritis	9 F 4 M	MoP	yes	Pain swelling, mass	One stage Excision pseudotumor and revision the nodular femoral head tocerami	N.R	N.R
Caleb W. Grote et al (2018) [1]	Case presentation and literature review	1	58	4	F	MoM	N.R		Compresision external iliac vaiin	One stage (revision THA)	no	Complete Resolution symptomsafter 4 years
Hart et al. [44]	Case series	34	N.R	34	N.R	N.R	N.R		17Symptomatic 17 asymptomatic	N.R	N.R	N.R
Sagoo et al. (2020) [45]	Case report	1	53	11	HIV	M	MoM		Severe Chronic abdominal pain syntomatic psoas fluid collection communicate with MoM prosthesis	One stage	No	Minimal pain
Mossavaghi et al. (2021) [58]	Case report	1	67	7	osteoarthritis	M	CoC		Aseptic loosening pain	One stage	no	HHS pre 45 post 67 Good functional
Filer et al. (2021) [10]	Case report	1	62	14	N.R	F	MoM		Haemorragic pseudotumor	Revision Hip (Cer-Pol) no excision pseudotumor	no	Regression of the pseudotumor, Resolution of symptoms
Rodriguetz et al (2022) [46]	Case report	1	71	13	N.R	M	Cer-Cer		Femoral nerve compression	One stage	no	no pain, paresthesia and neuralgia have been resolved
Huang et al. (2022) [48]	Case report	1	73	7	N.R	F	Cer-Cer		pain	One stage	dislocation	HHS 90 asymptomatic
Present study	Retrospective study e revision of literature	21	69 (range 50-82)	9,86 (range 1-20)	DM, IPA, stoke, arthritis , obesity, dysplasia, ..	13 F, 8 M	11 MoP 7 MoM 2 CoC 1 CoP	yes	Swelling, pain	**10 one stage 10 two stage 1 excision only**	5 maior (2 death) 3 deep infection 3 minor	**HHS pre 35 HHS post 75**

N.R.: not reported, M:male; F:female; MoM: Metal on metal; MoP:Metal on Polyetilene; CoC: Ceramic on Ceramic; CoM: Ceramic on Metal; THA: total Hip Artroplasty, HHS: Harry Hip Scores, DM: Diabetes Mellitus; IPA: Hypertension
