# Supplementary material for: When Is a Two-Stage Surgical Procedure Indicated in the Treatment of Pseudotumors of the Hip? A Retrospective Study of 21 Cases and a Review of the Literature
Source: J Clin Med. 2024 Jan 31;13(3):815. doi: 10.3390/jcm13030815 (PMC10856725; doi:10.3390/jcm13030815)
Supplement: Supplementary file 1 [file jcm-13-00815-s001.zip › Supplementary_material/Table_S1.pptx]

## Slide 1
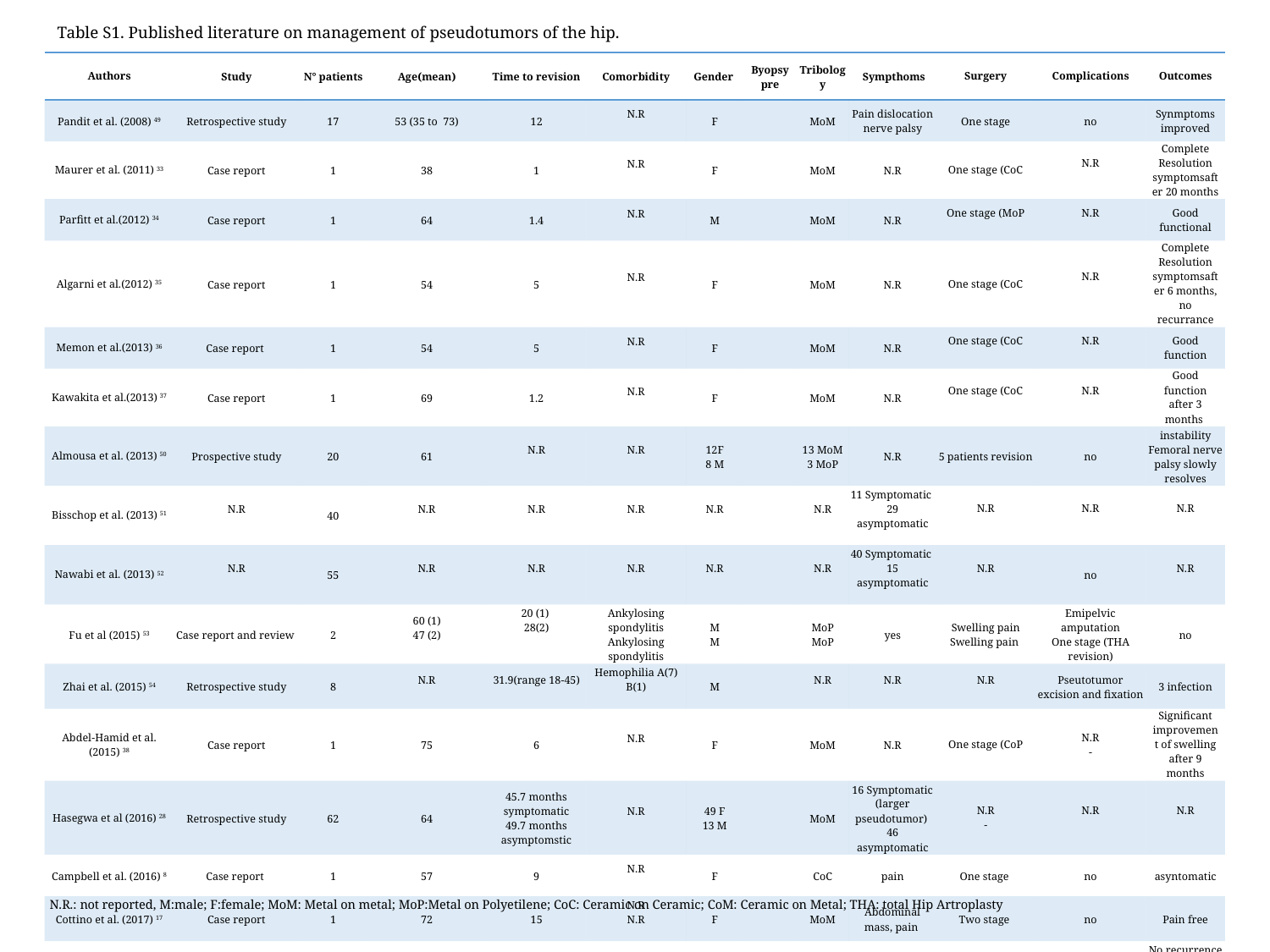

Table S1. Published literature on management of pseudotumors of the hip.
| Authors | Study | N° patients | Age(mean) | Time to revision | Comorbidity | Gender | Byopsy pre | Tribology | Sympthoms | Surgery | Complications | Outcomes |
| --- | --- | --- | --- | --- | --- | --- | --- | --- | --- | --- | --- | --- |
| Pandit et al. (2008) 49 | Retrospective study | 17 | 53 (35 to 73) | 12 | N.R | F | | MoM | Pain dislocation nerve palsy | One stage | no | Synmptoms improved |
| Maurer et al. (2011) 33 | Case report | 1 | 38 | 1 | N.R | F | | MoM | N.R | One stage (CoC | N.R | Complete Resolution symptomsafter 20 months |
| Parfitt et al.(2012) 34 | Case report | 1 | 64 | 1.4 | N.R | M | | MoM | N.R | One stage (MoP | N.R | Good functional |
| Algarni et al.(2012) 35 | Case report | 1 | 54 | 5 | N.R | F | | MoM | N.R | One stage (CoC | N.R | Complete Resolution symptomsafter 6 months, no recurrance |
| Memon et al.(2013) 36 | Case report | 1 | 54 | 5 | N.R | F | | MoM | N.R | One stage (CoC | N.R | Good function |
| Kawakita et al.(2013) 37 | Case report | 1 | 69 | 1.2 | N.R | F | | MoM | N.R | One stage (CoC | N.R | Good function after 3 months |
| Almousa et al. (2013) 50 | Prospective study | 20 | 61 | N.R | N.R | 12F 8 M | | 13 MoM 3 MoP | N.R | 5 patients revision | no | instability Femoral nerve palsy slowly resolves |
| Bisschop et al. (2013) 51 | N.R | 40 | N.R | N.R | N.R | N.R | | N.R | 11 Symptomatic 29 asymptomatic | N.R | N.R | N.R |
| Nawabi et al. (2013) 52 | N.R | 55 | N.R | N.R | N.R | N.R | | N.R | 40 Symptomatic 15 asymptomatic | N.R | no | N.R |
| Fu et al (2015) 53 | Case report and review | 2 | 60 (1) 47 (2) | 20 (1) 28(2) | Ankylosing spondylitis Ankylosing spondylitis | M M | | MoP MoP | yes | Swelling pain Swelling pain | Emipelvic amputation One stage (THA revision) | no |
| Zhai et al. (2015) 54 | Retrospective study | 8 | N.R | 31.9(range 18-45) | Hemophilia A(7) B(1) | M | | N.R | N.R | N.R | Pseutotumor excision and fixation | 3 infection |
| Abdel-Hamid et al.(2015) 38 | Case report | 1 | 75 | 6 | N.R | F | | MoM | N.R | One stage (CoP | N.R - | Significant improvement of swelling after 9 months |
| Hasegwa et al (2016) 28 | Retrospective study | 62 | 64 | 45.7 months symptomatic 49.7 months asymptomstic | N.R | 49 F 13 M | | MoM | 16 Symptomatic (larger pseudotumor) 46 asymptomatic | N.R - | N.R | N.R |
| Campbell et al. (2016) 8 | Case report | 1 | 57 | 9 | N.R | F | | CoC | pain | One stage | no | asyntomatic |
| Cottino et al. (2017) 17 | Case report | 1 | 72 | 15 | N.R N.R | F | | MoM | Abdominal mass, pain | Two stage | no | Pain free |
| Blau et al. (2017) 12 | Case report | 1 | 69 | 7 | N.R. | F | | CoM | Hip pain 8 elevated serum cobalt | N.R | no | No recurrence decresed level cobalt chromium |
N.R.: not reported, M:male; F:female; MoM: Metal on metal; MoP:Metal on Polyetilene; CoC: Ceramic on Ceramic; CoM: Ceramic on Metal; THA: total Hip Artroplasty

## Slide 2
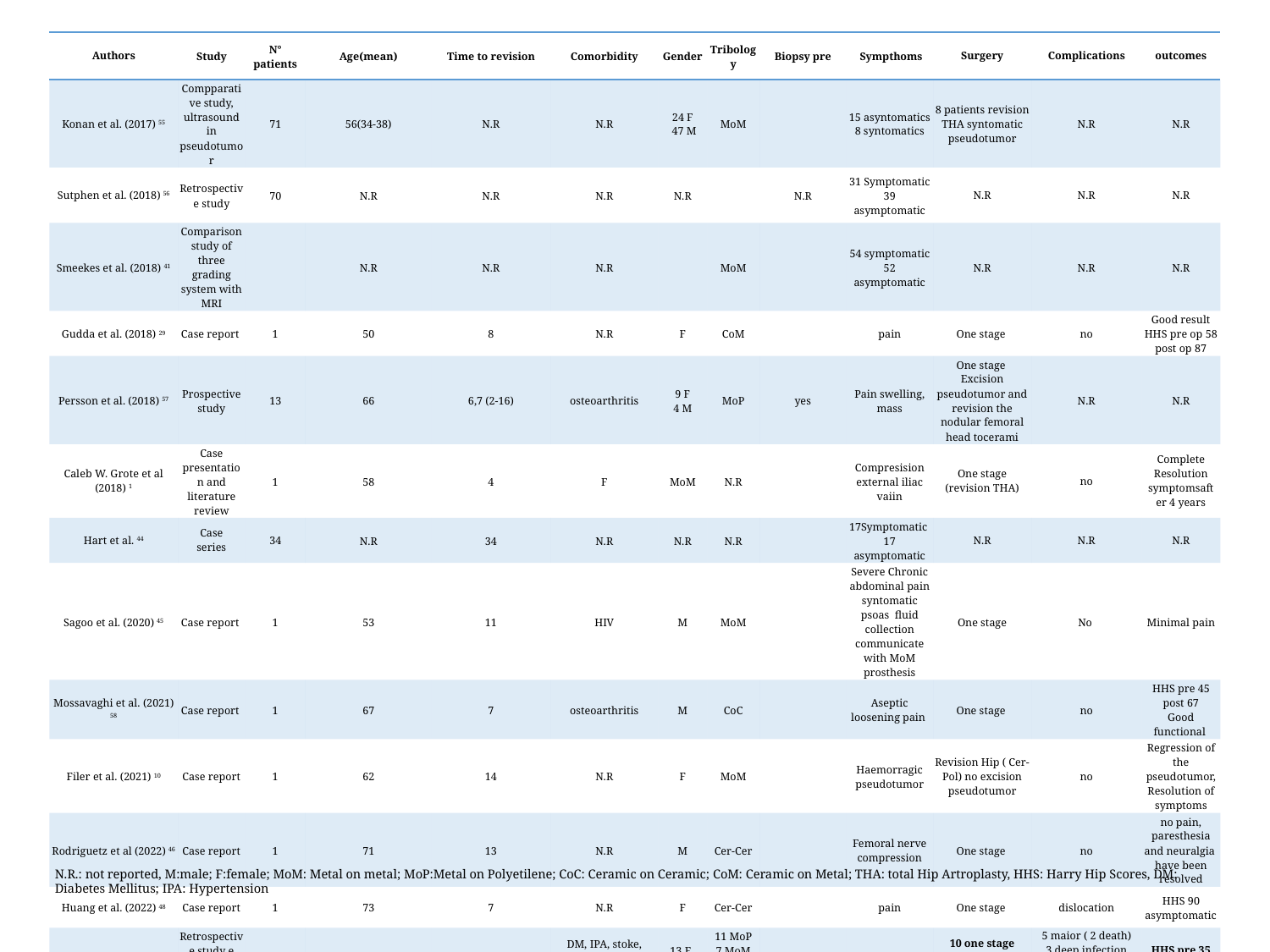

| Authors | Study | N° patients | Age(mean) | Time to revision | Comorbidity | Gender | Tribology | Biopsy pre | Sympthoms | Surgery | Complications | outcomes |
| --- | --- | --- | --- | --- | --- | --- | --- | --- | --- | --- | --- | --- |
| Konan et al. (2017) 55 | Compparative study, ultrasound in pseudotumor | 71 | 56(34-38) | N.R | N.R | 24 F 47 M | MoM | | 15 asyntomatics 8 syntomatics | 8 patients revision THA syntomatic pseudotumor | N.R | N.R |
| Sutphen et al. (2018) 56 | Retrospective study | 70 | N.R | N.R | N.R | N.R | | N.R | 31 Symptomatic 39 asymptomatic | N.R | N.R | N.R |
| Smeekes et al. (2018) 41 | Comparison study of three grading system with MRI | | N.R | N.R | N.R | | MoM | | 54 symptomatic 52 asymptomatic | N.R | N.R | N.R |
| Gudda et al. (2018) 29 | Case report | 1 | 50 | 8 | N.R | F | CoM | | pain | One stage | no | Good result HHS pre op 58 post op 87 |
| Persson et al. (2018) 57 | Prospective study | 13 | 66 | 6,7 (2-16) | osteoarthritis | 9 F 4 M | MoP | yes | Pain swelling, mass | One stage Excision pseudotumor and revision the nodular femoral head tocerami | N.R | N.R |
| Caleb W. Grote et al (2018) 1 | Case presentation and literature review | 1 | 58 | 4 | F | MoM | N.R | | Compresision external iliac vaiin | One stage (revision THA) | no | Complete Resolution symptomsafter 4 years |
| Hart et al. 44 | Case series | 34 | N.R | 34 | N.R | N.R | N.R | | 17Symptomatic 17 asymptomatic | N.R | N.R | N.R |
| Sagoo et al. (2020) 45 | Case report | 1 | 53 | 11 | HIV | M | MoM | | Severe Chronic abdominal pain syntomatic psoas fluid collection communicate with MoM prosthesis | One stage | No | Minimal pain |
| Mossavaghi et al. (2021) 58 | Case report | 1 | 67 | 7 | osteoarthritis | M | CoC | | Aseptic loosening pain | One stage | no | HHS pre 45 post 67 Good functional |
| Filer et al. (2021) 10 | Case report | 1 | 62 | 14 | N.R | F | MoM | | Haemorragic pseudotumor | Revision Hip ( Cer-Pol) no excision pseudotumor | no | Regression of the pseudotumor, Resolution of symptoms |
| Rodriguetz et al (2022) 46 | Case report | 1 | 71 | 13 | N.R | M | Cer-Cer | | Femoral nerve compression | One stage | no | no pain, paresthesia and neuralgia have been resolved |
| Huang et al. (2022) 48 | Case report | 1 | 73 | 7 | N.R | F | Cer-Cer | | pain | One stage | dislocation | HHS 90 asymptomatic |
| Present study | Retrospective study e revision of literature | 21 | 69 (range 50-82) | 9,86 (range 1-20) | DM, IPA, stoke, arthritis , obesity, dysplasia, .. | 13 F, 8 M | 11 MoP 7 MoM 2 CoC 1 CoP | yes | Swelling, pain | 10 one stage 10 two stage 1 excision only | 5 maior ( 2 death) 3 deep infection 3 minor | HHS pre 35 HHS post 75 |
N.R.: not reported, M:male; F:female; MoM: Metal on metal; MoP:Metal on Polyetilene; CoC: Ceramic on Ceramic; CoM: Ceramic on Metal; THA: total Hip Artroplasty, HHS: Harry Hip Scores, DM: Diabetes Mellitus; IPA: Hypertension
