# Supplementary figures and images for: When Is a Two-Stage Surgical Procedure Indicated in the Treatment of Pseudotumors of the Hip? A Retrospective Study of 21 Cases and a Review of the Literature
Source: J Clin Med. 2024 Jan 31;13(3):815. doi: 10.3390/jcm13030815 (PMC10856725; doi:10.3390/jcm13030815)

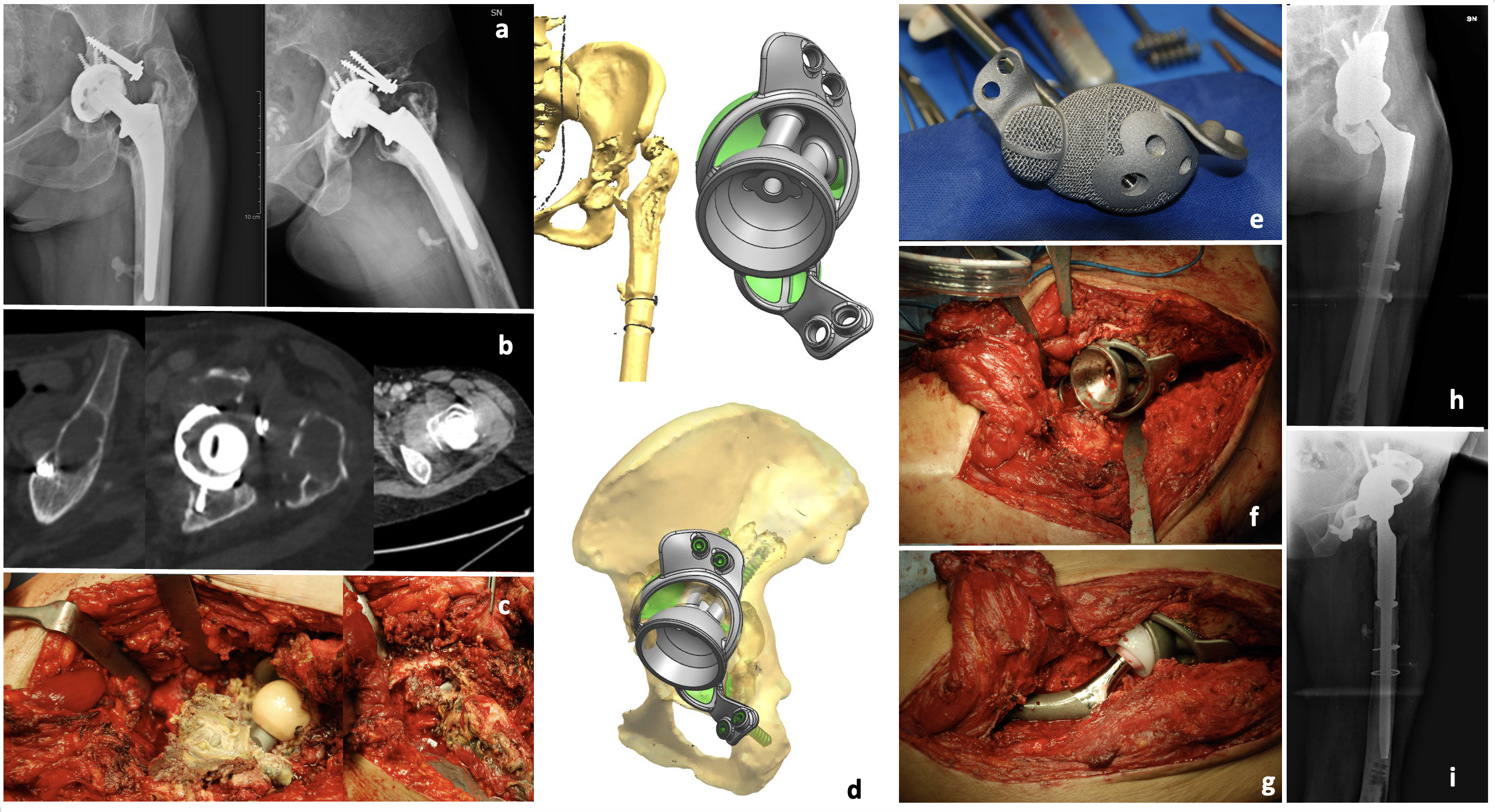

Supplement: Supplementary file 1 [file jcm-13-00815-s001.zip › Supplementary_material/Figure_S1.tiff]
